# Supplementary material for: Computational Analysis of Lifespan Experiment Reproducibility
Source: Front Genet. 2017 Jun 30;8:92. doi: 10.3389/fgene.2017.00092 (PMC5492194; doi:10.3389/fgene.2017.00092)

Supplementary Material

Title: Computational Analysis of Lifespan Experiment Reproducibility

**Michael Petrascheck* and Dana L. Miller**

*** Correspondence: pscheck@scripps.edu**

# Supplementary Figures and Tables

Tables showing the probability of detection for % increases in lifespan as a function of scoring frequency (Table 1), number of animals (Table 2) and for different number of animals as well as different distributions γ (Table 3).

**Supplementary table 1. EFFECTS OF SCORING FREQUENCY (LIVE/DEAD) ON POD**

**α=0.05**; **n= 100**

| %increase | ***2*** | ***4*** | ***6*** | ***8*** | ***10*** | ***12*** | ***14*** | ***16*** | ***18*** | ***20*** | ***22*** | ***24*** | ***26*** | ***28*** | ***30*** |
| --- | --- | --- | --- | --- | --- | --- | --- | --- | --- | --- | --- | --- | --- | --- | --- |
| d0.5 | 0.0731 | 0.1374 | 0.2471 | 0.3975 | 0.5584 | 0.7149 | 0.8412 | 0.9174 | 0.9654 | 0.9868 | 0.9945 | 0.9986 | 0.999 | 1 | 1 |
| d1 | 0.0729 | 0.1301 | 0.2444 | 0.3992 | 0.5576 | 0.7138 | 0.8364 | 0.9105 | 0.9639 | 0.9876 | 0.9953 | 0.9985 | 0.999 | 1 | 1 |
| d2 | 0.0674 | 0.1343 | 0.2387 | 0.3884 | 0.5409 | 0.7144 | 0.8359 | 0.9138 | 0.9617 | 0.985 | 0.9949 | 0.9983 | 0.9998 | 1 | 1 |
| d3 | 0.0709 | 0.1383 | 0.242 | 0.374 | 0.5363 | 0.6965 | 0.8173 | 0.9073 | 0.9611 | 0.9845 | 0.9938 | 0.9977 | 0.9996 | 1 | 1 |
| d5 | 0.0678 | 0.134 | 0.2237 | 0.3677 | 0.5212 | 0.6652 | 0.7941 | 0.8883 | 0.9491 | 0.9786 | 0.9939 | 0.9977 | 0.9995 | 0.9999 | 1 |
| d10 | 0.0634 | 0.115 | 0.187 | 0.3171 | 0.4464 | 0.592 | 0.7189 | 0.83 | 0.905 | 0.9521 | 0.977 | 0.9903 | 0.9966 | 0.9992 | 1 |
| d20 | 0.0551 | 0.0855 | 0.1224 | 0.1846 | 0.2564 | 0.3469 | 0.4364 | 0.5465 | 0.6323 | 0.7184 | 0.793 | 0.8588 | 0.9083 | 0.9474 | 0.9672 |

**α=0.05**; **n= 50**

| %increase | ***2*** | ***4*** | ***6*** | ***8*** | ***10*** | ***12*** | ***14*** | ***16*** | ***18*** | ***20*** | ***22*** | ***24*** | ***26*** | ***28*** | ***30*** |
| --- | --- | --- | --- | --- | --- | --- | --- | --- | --- | --- | --- | --- | --- | --- | --- |
| d0.5 | 0.0618 | 0.0964 | 0.1429 | 0.2225 | 0.315 | 0.42 | 0.5342 | 0.6503 | 0.7519 | 0.8292 | 0.8905 | 0.9372 | 0.9621 | 0.9818 | 0.9921 |
| d1 | 0.0657 | 0.1013 | 0.1432 | 0.2227 | 0.3165 | 0.4129 | 0.5349 | 0.6593 | 0.7537 | 0.8352 | 0.8891 | 0.9379 | 0.9616 | 0.9804 | 1 |
| d2 | 0.0614 | 0.0973 | 0.1414 | 0.2285 | 0.3129 | 0.420 | 0.5322 | 0.637 | 0.7398 | 0.8273 | 0.8857 | 0.9314 | 0.96 | 0.9784 | 0.9989 |
| d3 | 0.0647 | 0.0905 | 0.1457 | 0.2079 | 0.3067 | 0.4059 | 0.5199 | 0.6338 | 0.7431 | 0.8288 | 0.8855 | 0.9268 | 0.9582 | 0.974 | 0.9888 |
| d5 | 0.0623 | 0.0816 | 0.13 | 0.2131 | 0.2899 | 0.3914 | 0.4907 | 0.6111 | 0.7119 | 0.7951 | 0.8648 | 0.9144 | 0.9477 | 0.9711 | 0.9849 |
| d10 | 0.0539 | 0.081 | 0.1196 | 0.1729 | 0.2456 | 0.3311 | 0.429 | 0.5299 | 0.6313 | 0.7166 | 0.7972 | 0.8516 | 0.908 | 0.9399 | 0.96 |
| d20 | 0.0513 | 0.0604 | 0.0825 | 0.1124 | 0.1419 | 0.1812 | 0.241 | 0.2967 | 0.3578 | 0.4161 | 0.4926 | 0.5706 | 0.6337 | 0.6971 | 0.7587 |

**Supplementary tables 2. EFFECTS OF THE NUMBER OF ANIMALS N ON POD**

**α=0.05**; **scoring interval: 2 days**

| %increase | ***2*** | ***4*** | ***6*** | ***8*** | ***10*** | ***12*** | ***14*** | ***16*** | ***18*** | ***20*** | ***22*** | ***24*** | ***26*** | ***28*** | ***30*** |
| --- | --- | --- | --- | --- | --- | --- | --- | --- | --- | --- | --- | --- | --- | --- | --- |
| n= 20 | 0.0648 | 0.0755 | 0.0924 | 0.1205 | 0.1591 | 0.1951 | 0.2456 | 0.2921 | 0.3593 | 0.4236 | 0.4923 | 0.5599 | 0.6303 | 0.6865 | 0.741 |
| n= 50 | 0.0643 | 0.0908 | 0.1376 | 0.209 | 0.3093 | 0.4079 | 0.5148 | 0.6321 | 0.7329 | 0.8216 | 0.8741 | 0.9222 | 0.9599 | 0.9766 | 0.9885 |
| n= 100 | 0.0775 | 0.1362 | 0.2398 | 0.3771 | 0.5443 | 0.6972 | 0.8211 | 0.9048 | 0.9608 | 0.9833 | 0.9948 | 0.9973 | 0.9994 | 0.9998 | 1 |
| n= 150 | 0.0807 | 0.1757 | 0.3257 | 0.5251 | 0.7206 | 0.855 | 0.9402 | 0.9795 | 0.9955 | 0.9988 | 0.9999 | 1 | 1 | 1 | 1 |
| n= 200 | 0.0936 | 0.214 | 0.427 | 0.6935 | 0.8363 | 0.9434 | 0.9849 | 0.9966 | 0.9995 | 1 | 1 | 1 | 1 | 1 | 1 |

**α=0.01**; **scoring interval: 2 days**

| %increase | ***2*** | ***4*** | ***6*** | ***8*** | ***10*** | ***12*** | ***14*** | ***16*** | ***18*** | ***20*** | ***22*** | ***24*** | ***26*** | ***28*** | ***30*** |
| --- | --- | --- | --- | --- | --- | --- | --- | --- | --- | --- | --- | --- | --- | --- | --- |
| n= 20 | 0.0133 | 0.0177 | 0.0259 | 0.0369 | 0.0564 | 0.0755 | 0.0925 | 0.1335 | 0.1701 | 0.2122 | 0.2676 | 0.3144 | 0.3819 | 0.4433 | 0.5082 |
| n= 50 | 0.0159 | 0.025 | 0.0486 | 0.0814 | 0.1326 | 0.2035 | 0.2962 | 0.4019 | 0.5219 | 0.6346 | 0.7267 | 0.8075 | 0.8764 | 0.9209 | 0.9584 |
| n= 100 | 0.0187 | 0.0436 | 0.0965 | 0.1809 | 0.3155 | 0.4777 | 0.6296 | 0.7689 | 0.8772 | 0.9372 | 0.9739 | 0.9912 | 0.9978 | 0.9992 | 0.9998 |
| n= 150 | 0.0218 | 0.0621 | 0.1593 | 0.3002 | 0.4989 | 0.6896 | 0.8452 | 0.9402 | 0.9817 | 0.9933 | 0.9985 | 0.9997 | 0.9999 | 1 | 1 |
| n= 200 | 0.0261 | 0.0885 | 0.2234 | 0.4294 | 0.6585 | 0.8388 | 0.9397 | 0.9854 | 0.9975 | 0.9992 | 1 | 1 | 1 | 1 | 1 |

**α=0.001**; **scoring interval: 2 days**

| %increase | ***2*** | ***4*** | ***6*** | ***8*** | ***10*** | ***12*** | ***14*** | ***16*** | ***18*** | ***20*** | ***22*** | ***24*** | ***26*** | ***28*** | ***30*** |
| --- | --- | --- | --- | --- | --- | --- | --- | --- | --- | --- | --- | --- | --- | --- | --- |
| n= 20 | 0.0017 | 0.0018 | 0.0038 | 0.0067 | 0.0097 | 0.0149 | 0.0211 | 0.0327 | 0.0466 | 0.0653 | 0.082 | 0.1146 | 0.1459 | 0.1919 | 0.2256 |
| n= 50 | 0.0019 | 0.0042 | 0.0093 | 0.0174 | 0.0352 | 0.0645 | 0.0981 | 0.1656 | 0.2381 | 0.3373 | 0.4341 | 0.5527 | 0.6586 | 0.7484 | 0.823 |
| n= 100 | 0.0023 | 0.0067 | 0.0224 | 0.0527 | 0.1161 | 0.2168 | 0.3502 | 0.5119 | 0.6591 | 0.7865 | 0.8885 | 0.9485 | 0.9792 | 0.9931 | 0.9973 |
| n= 150 | 0.0036 | 0.013 | 0.0422 | 0.1095 | 0.2416 | 0.4112 | 0.6215 | 0.7877 | 0.9046 | 0.9646 | 0.989 | 0.9974 | 0.9993 | 0.9999 | 0.9999 |
| n= 200 | 0.0036 | 0.0198 | 0.0726 | 0.1788 | 0.3795 | 0.5998 | 0.808 | 0.9213 | 0.9797 | 0.9969 | 0.9987 | 1 | 1 | 1 | 1 |

**Supplementary tables 3. EFFECTS OF THE NUMBER OF ANIMALS N ON POD COMPARING DIFFERENT DISTRIBUTION**

**α=0.05**; **scoring interval: 2 days**

| gamma | 0.1275 | 0.124 | 0.1205 | 0.117 | 0.1135 | 0.11 | 0.1065 | 0.103 | 0.0995 | 0.096 | 0.0925 | 0.089 | 0.0855 |
| --- | --- | --- | --- | --- | --- | --- | --- | --- | --- | --- | --- | --- | --- |
| %increase | ***2*** | ***4*** | ***6*** | ***8*** | ***10*** | ***12*** | ***15*** | ***17*** | ***20*** | ***23*** | ***26*** | ***29*** | ***32*** |
| n= 20 | 0.055 | 0.0694 | 0.0954 | 0.1264 | 0.17 | 0.2259 | 0.2939 | 0.3715 | 0.4438 | 0.5286 | 0.6067 | 0.6913 | 0.761 |
| n= 50 | 0.063 | 0.0972 | 0.1631 | 0.245 | 0.3685 | 0.4979 | 0.6363 | 0.757 | 0.8417 | 0.9165 | 0.9548 | 0.9797 | 0.991 |
| n= 100 | 0.0703 | 0.1474 | 0.2769 | 0.4509 | 0.6456 | 0.8008 | 0.9148 | 0.964 | 0.9893 | 0.9968 | 0.9996 | 0.9999 | 1 |
| n= 150 | 0.0868 | 0.1907 | 0.4016 | 0.6168 | 0.8122 | 0.9345 | 0.9812 | 0.997 | 0.9996 | 0.9999 | 1 | 1 | 1 |
| n= 200 | 0.1007 | 0.251 | 0.4985 | 0.7469 | 0.9109 | 0.9801 | 0.9965 | 0.9998 | 1 | 1 | 1 | 1 | 1 |

**α=0.01**; **scoring interval: 2 days**

| gamma | 0.1275 | 0.124 | 0.1205 | 0.117 | 0.1135 | 0.11 | 0.1065 | 0.103 | 0.0995 | 0.096 | 0.0925 | 0.089 | 0.0855 |
| --- | --- | --- | --- | --- | --- | --- | --- | --- | --- | --- | --- | --- | --- |
| %increase | ***2*** | ***4*** | ***6*** | ***8*** | ***10*** | ***12*** | ***15*** | ***17*** | ***20*** | ***23*** | ***26*** | ***29*** | ***32*** |
| n= 20 | 0.013 | 0.0157 | 0.0201 | 0.0332 | 0.0512 | 0.0739 | 0.1145 | 0.1523 | 0.2032 | 0.2752 | 0.3366 | 0.4181 | 0.4951 |
| n= 50 | 0.0144 | 0.0239 | 0.0505 | 0.0964 | 0.1584 | 0.2511 | 0.3838 | 0.5081 | 0.6431 | 0.7516 | 0.842 | 0.915 | 0.954 |
| n= 100 | 0.0168 | 0.0492 | 0.1114 | 0.2294 | 0.3865 | 0.5764 | 0.7533 | 0.8835 | 0.9484 | 0.985 | 0.9953 | 0.9993 | 1 |
| n= 150 | 0.0207 | 0.0725 | 0.1878 | 0.3705 | 0.6057 | 0.8059 | 0.929 | 0.9804 | 0.9962 | 0.9995 | 0.9998 | 1 | 1 |
| n= 200 | 0.0244 | 0.1036 | 0.2619 | 0.5198 | 0.764 | 0.9205 | 0.9821 | 0.9974 | 0.9997 | 1 | 1 | 1 | 1 |

**α=0.001**; **scoring interval: 2 days**

| gamma | 0.1275 | 0.124 | 0.1205 | 0.117 | 0.1135 | 0.11 | 0.1065 | 0.103 | 0.0995 | 0.096 | 0.0925 | 0.089 | 0.0855 |
| --- | --- | --- | --- | --- | --- | --- | --- | --- | --- | --- | --- | --- | --- |
| %increase | ***2*** | ***4*** | ***6*** | ***8*** | ***10*** | ***12*** | ***15*** | ***17*** | ***20*** | ***23*** | ***26*** | ***29*** | ***32*** |
| n= 20 | 0.0007 | 0.005 | 0.0026 | 0.0043 | 0.0085 | 0.0106 | 0.0206 | 0.0336 | 0.05 | 0.0716 | 0.1066 | 0.1514 | 0.1964 |
| n= 50 | 0.0018 | 0.0035 | 0.0094 | 0.0183 | 0.0389 | 0.0781 | 0.1289 | 0.2204 | 0.3375 | 0.4646 | 0.6057 | 0.7191 | 0.8129 |
| n= 100 | 0.0026 | 0.0079 | 0.0263 | 0.0614 | 0.1541 | 0.2852 | 0.4683 | 0.6588 | 0.812 | 0.9133 | 0.9697 | 0.9889 | 0.9976 |
| n= 150 | 0.0026 | 0.0079 | 0.0517 | 0.1417 | 0.3158 | 0.5355 | 0.7584 | 0.901 | 0.9713 | 0.9937 | 0.9992 | 1 | 1 |
| n= 200 | 0.0048 | 0.0243 | 0.0831 | 0.2346 | 0.485 | 0.7518 | 0.9106 | 0.9788 | 0.9974 | 0.9995 | 0.9999 | 1 | 1 |

## Supplementary Figures

**Supplementary Figure 1. EFFECTS OF SCORING FREQUENCY (LIVE/DEAD) ON POD**

Plots representing the data in Supplementary Table 1

**Supplementary Figure 1.** Each plot shows probability of detection as a function of % increase in lifespan for different scoring frequencies. Left, n=50 (left), Right, n=100. The significance level α remains constant at 0.05. In these plots increases in lifespan were simulated by modulating G of the gompertz equation.

**Supplementary Figure 2. EFFECTS OF THE NUMBER OF ANIMALS N ON POD**

Plots representing the data in Supplementary Table 2

**Supplementary Figure 2.** Each plot shows probability of detection as a function of % increase in lifespan for different number of animals (n). The scoring frequency is kept at 2 days. Plots differ in the significance level α. In these plots increases in lifespan were simulated by modulating G of the gompertz equation

**Supplementary Figure 3. EFFECTS OF THE NUMBER OF ANIMALS N ON POD COMPARING DIFFERENT DISTRIBUTION**

Plots representing the data in Supplementary Table 3

**Supplementary Figure 3.** Each plot shows probability of detection as a function of % increase in lifespan for different number of animals (n). The scoring frequency is kept at 2 days. Plots differ in the significance level α. In these plots increases in lifespan were simulated by modulating γ rather than G.


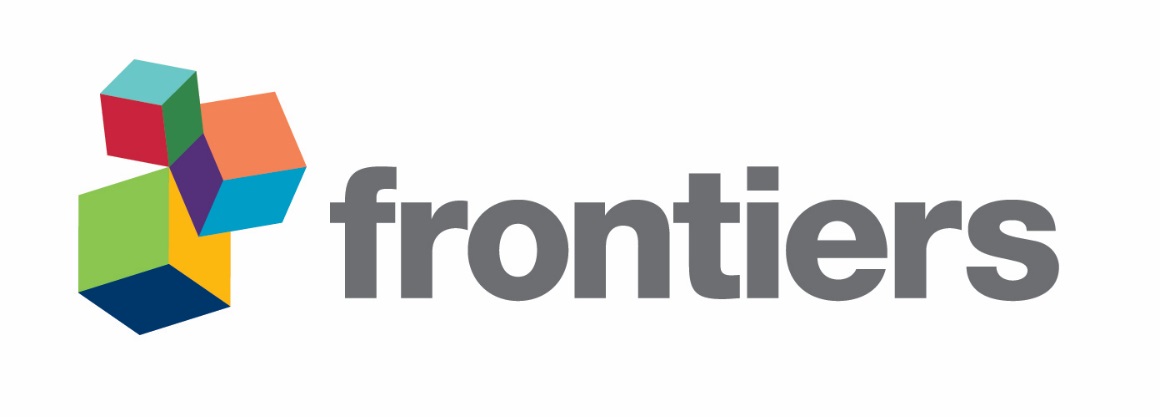

Supplement: Supplementary file 1 [file DataSheet1.docx]
